# Habitat characteristics and animal management factors associated with habitat use by bottlenose dolphins in zoological environments

**DOI:** 10.1371/journal.pone.0252010

**Published:** 2021-08-30

**Authors:** Lisa K. Lauderdale, K. Alex Shorter, Ding Zhang, Joaquin Gabaldon, Jill D. Mellen, Michael T. Walsh, Douglas A. Granger, Lance J. Miller

**Affiliations:** 1 Conservation Science and Animal Welfare Research, Chicago Zoological Society–Brookfield Zoo, Brookfield, IL, United States of America; 2 Department of Mechanical Engineering, University of Michigan, Ann Arbor, MI, United States of America; 3 Robotics Institute, University of Michigan, Ann Arbor, MI, United States of America; 4 Biology Department, Portland State University, Portland, OR, United States of America; 5 Department of Comparative, Diagnostic & Population Medicine, College of Veterinary Medicine, University of Florida, Gainesville, FL, United States of America; 6 Institute for Interdisciplinary Salivary Bioscience Research, University of California, Irvine, CA, United States of America; Institute of Deep-sea Science and Engineering, Chinese Academy of Sciences, CHINA

## Abstract

The way an animal uses its habitat can serve as an indicator of habitat appropriateness for the species and individuals. Bottlenose dolphins (*Tursiops truncatus* and *Tursiops aduncus*) in accredited zoos and aquariums experience a range of habitat types and management programs that provide opportunities for dolphins to engage in species-appropriate behaviors and potentially influence their individual and group welfare. Data in the present study were collected as part of a larger study titled “Towards understanding the welfare of cetaceans in zoos and aquariums” (colloquially called the Cetacean Welfare Study). Non-invasive bio-logging devices (Movement Tags) recorded the diving behavior and vertical habitat movements of 60 bottlenose dolphins at 31 zoos and aquariums that were accredited by the Alliance for Marine Mammal Parks and Aquariums and/or the Association of Zoos & Aquariums. Bottlenose dolphins wore a Movement Tag one day per week for two five-week data collection periods. Demographic variables, environmental enrichment programs, training programs, and habitat characteristics were associated with habitat usage. Longer dive durations and use of the bottom third of the habitat were associated with higher enrichment program index values. Dolphins receiving new enrichment on a monthly/weekly schedule also used the bottom third of the habitat more often than those receiving new enrichment on a yearly/year+ schedule. Dolphins that were managed in a group that was split into smaller subgroups during the day and were reunited into one group at night spent less time in the top third of the habitat than those who remained in a single group with consistent members at all times. Dolphins that were managed as subgroups with rotating members but were never united as one group spent less time in the bottom third of the habitat than those who remained in a single group with consistent members at all times. Taken together, the results suggested that management practices, such as enrichment and training programs, played a greater role in how dolphins interacted with their environment relative to the physical characteristics of the habitat.

## Introduction

Modern zoological habitats are designed to maintain, enhance, and promote animal welfare. Positive welfare has been associated with habitats that are of an appropriate size, give animals access to multiple areas, and include environmental enrichment [1–3]. Examining how animals use their habitats can serve as an indicator of their appropriateness for the species and individuals [4,5]. Larger enclosure sizes have been positively correlated with increased locomotion and greater distance traveled for several terrestrial species [6]. However, continued expansion of size does not always lead to a corresponding increase in locomotion for all species. When chimpanzees (*Pan troglodytes*) were transferred between habitats above the National Institutes of Health recommended size, there were no changes in locomotion rates despite the new enclosure being smaller [7]. Following these moves, increases in behavioral diversity were observed, suggesting that the novelty of the post-transfer environment may have been enriching. In addition, many species show preferences for certain parts of their habitats based on biological relevance and resource availability [8,9]. For example, female hippos (*Hippopotamus amphibius*) show a preference for aggregating in water with depths of 0.6–1.0 m where they are able to sleep while standing [8]. African wild dogs (*Lycaon pictus)* and African elephants (*Loxodonta africana*) both show a preference for occupying spaces where high-value resources, such as food, are commonly supplied [4,10].

Management practices, such as food dispersion and adding environmental enrichment, can be used to influence habitat usage. Environmental enrichment (i.e., enhancing an animal’s environment through the addition of stimuli designed to promote species-appropriate behavior) can encourage animals to use certain parts of their habitat. For example, interaction with submerged enrichment increases the bottom third habitat usage by bottlenose dolphins (*Tursiops truncatus*) [11]. In other species, the introduction of novel scents increases habitat utilization and activity [12,13]. Enriched areas of habitats are associated with increases in social interactions, activity, and foraging behaviors as well as a decrease in stereotypic behaviors [3,14,15]. Similarly, provisioning of foraging enrichment increases the rate of exploration and a decrease in stereotypic behaviors [16–18].

Bottlenose dolphins in accredited zoos and aquariums experience a range of habitat types and management programs that are designed to provide opportunities for dolphins to engage in species-appropriate behaviors. However, further research is necessary to understand the relationships among habitat features, management programs, and habitat use. In the wild, dolphins in waterways near Sarasota Bay, Florida, USA reside in habitats up to 11 m deep [19,20]. Most sightings occur in areas that are less than 2–3 m deep. Dolphins use deeper areas for feeding when fish are not available in shallower areas [21,22]. Off the coast of southeast Queensland, Australia, resident bottlenose dolphins spend two-thirds of their time within 5 m of the surface [23].

Under professional care, it is presumed that both length and depth of habitats impact behavior. Relatively larger habitats have been associated with higher swimming rates and reduced aggression when one area of the habitat is available at a time [24–26]. When areas with depths of 3.96, 5.49, and 8.23 m were available, bottlenose dolphins chose to use the moderate and shallow depth areas 68% and 30% of the time, respectively [27]. Bassos and Wells [24] found that the horizontal dimension of the habitat was more related to positive (i.e., non-stereotypic) behaviors than any other dimension (i.e., depth or width). Similarly, when compared with circular habitats, oblong habitats promoted more successful nursing of killer whale (*Orcinus orca*) calves, presumably because it allows for longer, uninterrupted periods of slow fluking and gliding bouts during which nursing occurs [28].

In the present study, Movement Tags (MTags) were used to record the fine-scale movements of bottlenose dolphins. This data collection method required minimal hardware setup relative to camera/tag-based methods [29–31], thus making it preferable for a multi-facility study. MTags were used to examine how habitat characteristics and management practices were associated with diving behavior and vertical habitat usage. Understanding how bottlenose dolphins use their vertical environment could aid in future welfare and management related decisions and habitat design.

## Materials and methods

### Ethics statement

This study was authorized by each participating zoo and aquarium and, where applicable, was reviewed and approved by research committees. In addition, the U.S. Navy Marine Mammal Program Institutional Animal Care and Use Committee reviewed and approved the project #123–2017.

### Subjects and facilities

The present findings are part of a larger project entitled “Towards Understanding the Welfare of Cetaceans in Zoos and Aquariums” (colloquially called the Cetacean Welfare Study). Zoos and aquariums that were accredited in 2017 by the Alliance for Marine Mammal Parks and Aquariums and the Association of Zoos & Aquariums were eligible for participation in this portion of the larger Cetacean Welfare Study provided they cared for common bottlenose dolphins or Indo-Pacific bottlenose dolphins (*Tursiops aduncus*). Two animals from each participating facility were selected using a semi-random sampling design to create a balanced representation of the study population. For this study, data were collected from a total of 65 dolphins at 35 facilities. Participating facilities were located in Bermuda (n = 1), Hong Kong (n = 1), Jamaica (n = 2), Mexico (n = 15), Singapore (n = 1), Spain (n = 1), and the United States (n = 14). Dolphins lived in both professionally managed zoo/aquarium habitats and professionally managed ocean habitats. Professionally managed zoo/aquarium habitats were defined as fabricated habitats with or without exposure to weather patterns. Professionally managed ocean habitats were defined as cordoned off sections of coastal ocean, bays, lagoons, or waterways. S1 File_Appendix1_Lauderdale_HabitatUse provides the sex, age, and total minutes recorded outside of formal training sessions for all participants.

### Data collection

The MTag bio-logging device was used to monitor animal behavior for this study. MTags were 150 mm in length and 76 mm wide and were attached to the dolphins non-invasively approximately 20 cm behind the blowhole via four specially designed silicone suction cups. Prior to the study, the focal dolphins were trained to wear the MTags and they could be easily removed by the animal care staff at any time. The silicone suction cups did not result in any damage to the skin and similar bio-logging devices have been used extensively with wild dolphins prior to application in this study. The tag itself was designed with a hydrodynamic profile to reduce drag imparted to the animal [32].

**Fig 1:**
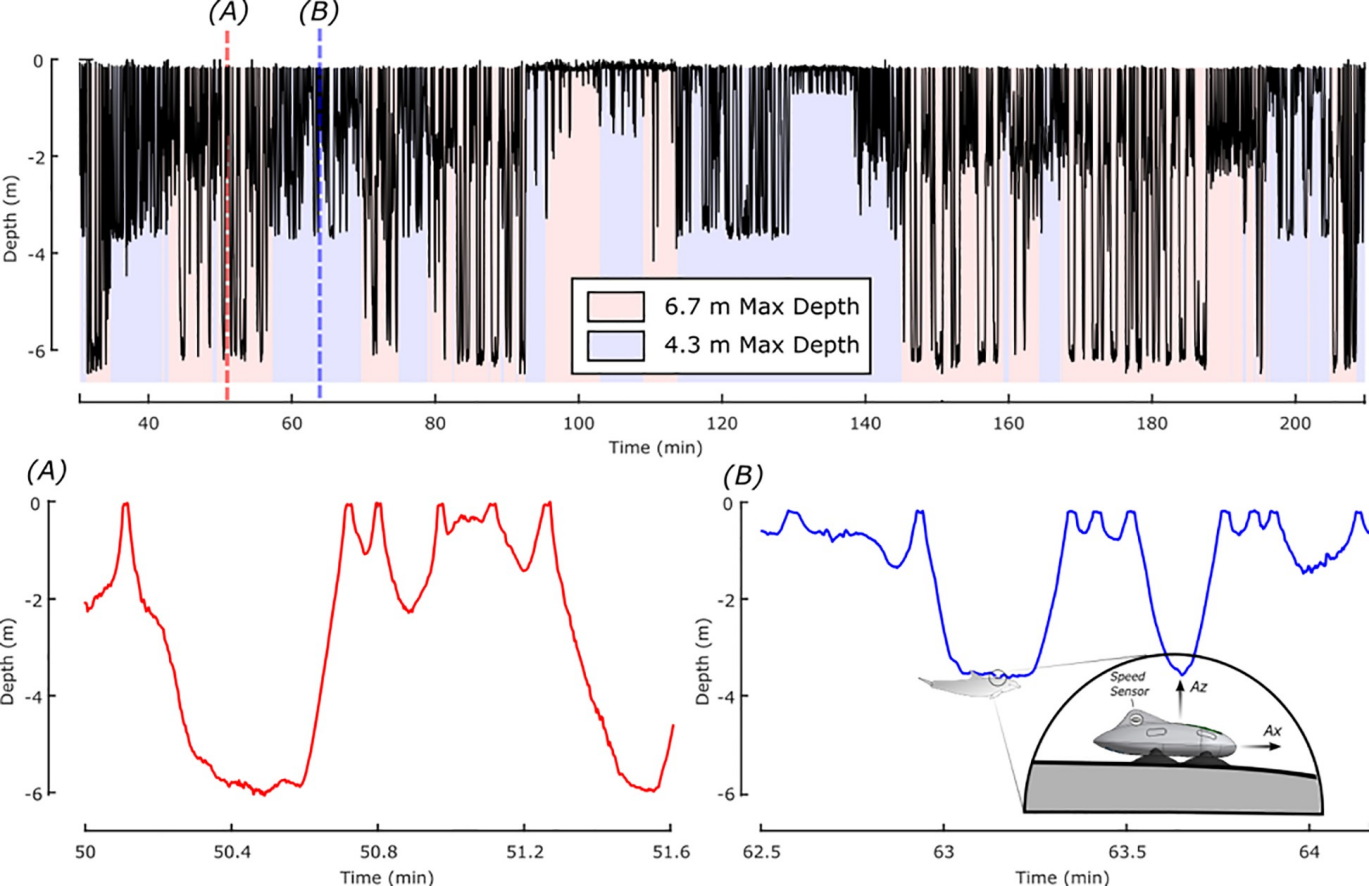
Pressure data. A representative section of pressure data recorded by the MTag in an environment with two areas of different maximum depths (Top). The plot is shaded red when the animal is in the 6.7 m deep area and blue when the animal is in the 4.3 m deep area. Details showing differences in the diving profiles in the two sections of the environment are shown in the bottom left (A, 6.7 m deep area) and right (B, 4.3 m deep area) figures, along with an image of the bio-logging tag used to collect the data (inset).

**Fig 2:**
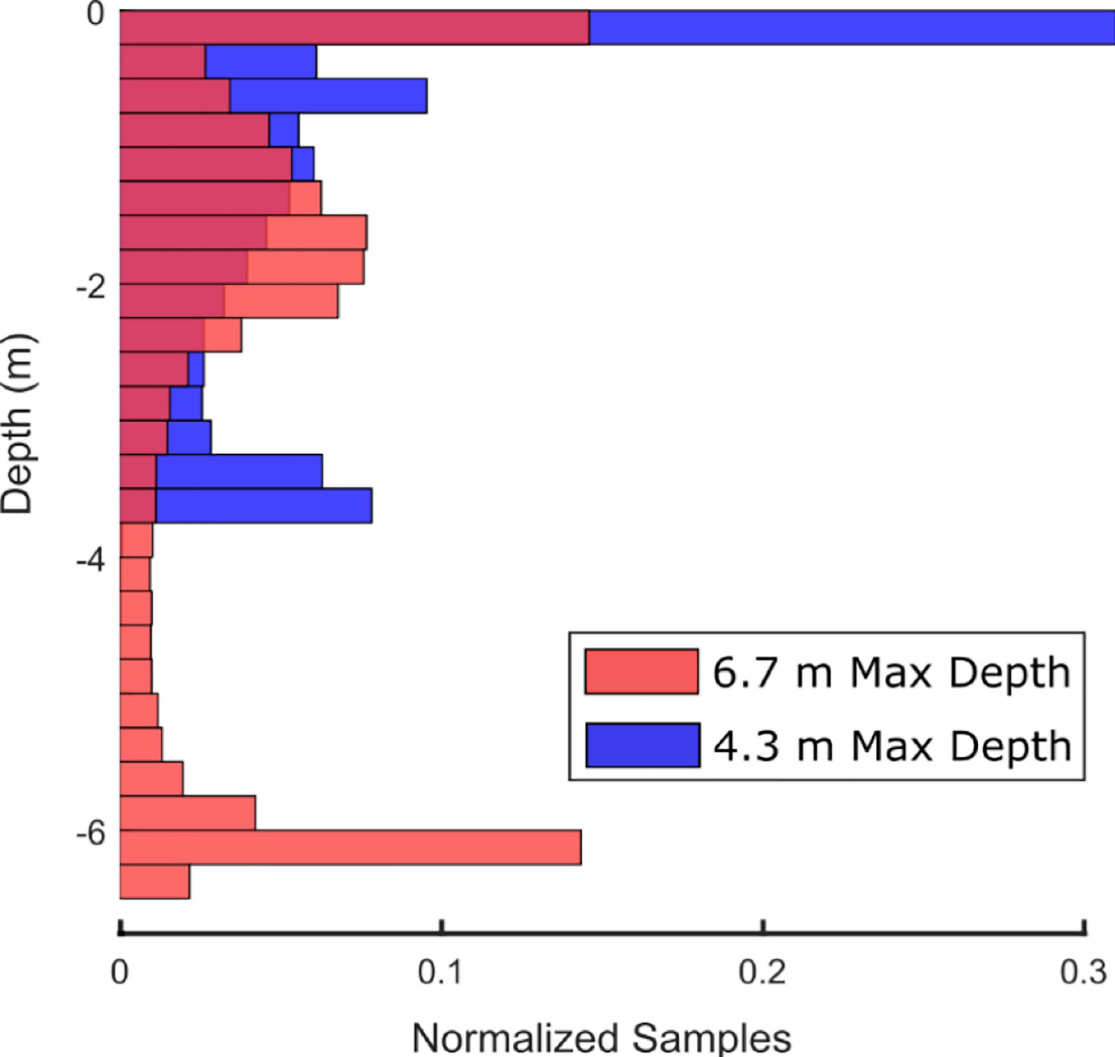
Representative depth data. Representative depth data (also used for Fig 1) taken from an individual animal at a facility with gated environments of different maximum depths. Depth data from one ~3.0 hr recording are shown for the section of the habitat with a maximum depth of 6.7 m in red and the section of the habitat with a maximum depth of 4.3 m in blue. Areas of overlap are indicated by the dark red color. The maximum depths achieved by the dolphin in each region were ~6.5 m and ~3.75 m, respectively. The sample counts for each histogram were normalized by the histogram’s total sample count (summing to 100% of the data) to ensure the comparison was valid.

The MTag’s electronics were designed for use with dolphins under professional care and were based on the Loggerhead Instruments OpenTag3 platform. The primary board of the OpenTag3 contained a 9-degree-of-freedom (DOF) inertial measurement unit (IMU; accelerometer, magnetometer, gyroscope) and sensors to measure environmental pressure and temperature. A 1-DOF Hall effect sensor was connected to the primary board to measure the rotations of a magnetic micro-turbine, mounted to an exterior fin on the tag, which provided an estimate of flow speed around the tagged animal. IMU data (i.e., accelerometer, magnetometer, and gyroscope) were sampled at 50 Hz (i.e., samples per second), and all other sensor data were sampled at 5 Hz. The speed sensor performance was characterized in Gabaldon *et al*. [33]. The board was encased in epoxy for waterproofing and mounted in a 3D-printed housing (stereolithography on a Formlabs Form 2™ printer). Fig 1 shows a sketch of the tag attached to a dolphin, along with a multi-hour representative section of pressure data recorded at one of the accredited facilities. This facility contains two primary types of habitats: one is 6.7 m deep and the other is 4.3 m deep. Example dives for the 6.7 m and 4.3 m deep habitats are included to demonstrate dolphin dive profiles in these representative habitats. Histograms of the depth distributions in each habitat for this individual data set are shown in Fig 2. As detailed in the Statistical Analysis section, data collected from the tag, pressure data in particular, played a critical role in characterizing environmental usage.

The Cetacean Welfare Study consisted of two data collection periods. The first data collection period was from July 2018 through November 2018 and the second period was from January 2019 through April 2019. Data were collected for one five-week period at each facility during both years. MTags were deployed Tuesdays and Fridays using an alternating schedule for the two dolphin participants at each location. Each dolphin wore the MTag throughout their normal daily activities once per week.

### Independent variables

Independent variables that were potentially relevant to animal welfare were selected to examine a variety of demographic variables, habitat characteristics, and management factors. The independent variables selected, and their definitions are presented in Table 1. Definitions of terms and methods for calculating the synthesized independent variables as well as environmental enrichment types are presented in Lauderdale *et al* [34].

**Table 1:** Independent variables included in the analysis.

Variable	Definition	Values	Type of Variable
***Demographic***			
Sex	Sex of the dolphin	Male/Female	Factor
Age	Age of the dolphin	Years	Covariate
***Environmental Enrichment***			
Enrichment Diversity Index	Enrichment diversity index was created using the Shannon diversity index on the mean number of days each enrichment is provided at the facility	Index	Covariate
Enrichment Program Index	Enrichment program index is a standardized factor score created with scores on frequency of enrichment program components used at the facility using a polychoric PCA	Index	Covariate
Night Time Enrichment	Mean number of nights in a week that enrichment was provided to the dolphins at the facility	Number of Nights	Covariate
Enrichment Schedule	Categorical value indicating how enrichment was scheduled at the facility	Predictable, Semi-Random, Random	Factor
Frequency of New Enrichment	Categorical frequency that a facility provided the dolphins with new types/forms of enrichment	Weekly/Monthly, Twice a Year, Yearly/Year+	Factor
***Training***			
Dolphin Presentations	Mean number of dolphin presentations an individual dolphin participated in each week	Mean Number of Presentations	Covariate
Interaction Programs	Mean number of dolphin interaction programs an individual dolphin participated in each week	Mean Number of Interactions	Covariate
Training Duration	Mean amount of time each dolphin interacted with an animal care professional for presentations, interaction programs, training sessions, research, or other training activities each week	Hours	Covariate
Maximum Number of Interaction Guests	Maximum number of participants allowed for an interaction program for that facility	Number of Participants	Covariate
Training Schedule	Categorical variable indicating if the training schedule for the dolphins at that facility was predictable or semi-predictable	Predictable, Semi-Predictable	Factor
***Habitat Characteristics***			
Day Time Spatial Experience	Proportionate volume of water the dolphin had access to based on the percentage of daytime hours spent in different habitats in each five-week data collection period	Megaliter	Covariate
Night Time Spatial Experience	Proportionate volume of water the dolphin had access to based on the percentage of night time hours spent in different habitats in each five-week data collection period	Megaliter	Covariate
24 Hour Spatial Experience	Proportionate volume of water the dolphin had access to based on the percentage of hours throughout the entire day spent in different habitats in each five-week data collection period	Megaliter	Covariate
Length	The maximum straight length in any direction across any habitat the dolphin had access to in each five-week data collection period	m	Covariate
Depth	The maximum depth for any habitat the dolphin had access to in each five-week data collection period	m	Covariate
Habitat Type	Categorical variable indicating the dolphin was in a professionally managed zoo/aquarium habitat or a professionally managed ocean habitat	Zoo/Aquarium, Ocean	Factor
Number of Habitats	Maximum number of habitats (different enclosures) dolphin had access to in daytime hours during each five-week data collection period	Number of Habitats	Covariate
Social Management	Categorical variable indicating the type of social management practice for a dolphin during each five-week data collection period	Same Group, Split/Reunited, Rotated Subgroups	Factor
Neighboring Conspecifics	Categorical variable indicating if the dolphin had visual and auditory access to other dolphins without possibility of physical contact during each five-week data collection period	No, Yes	Factor

### Statistical analysis

Swimming depth was estimated from pressure sensor data. Dolphins were classified as being in the top or bottom third of their habitat. The top third of the habitat was defined as the shallowest third of the water column based on the deepest depth available to the dolphin and the bottom third was defined as the deepest third of the water column available. Percent time spent in the top and bottom thirds of the habitat was calculated by dividing the total time spent in the focal portion of the water column by the total length of the MTag deployment. The dive duration was defined as the length of time between two surfacing events. Analyses were conducted in MATLAB 9.7.0 using custom scripts.

MTag data were selected during times in which dolphins were outside of formal training sessions to explore dolphin behavior throughout the day. Demographic and management characteristics were evaluated for their association with mean dive duration and top/bottom third habitat use. Due to the non-normal distribution of the data, statistical models were examined using generalized estimating equations (GEE). Additionally, GEE models do not require data transformation, which preserves the interpretability of the relationship between data and results [35,36].

In addition to wearing the bio-logging devices, the dolphins were video recorded three times per week for 25 minutes over the course of the five-week period [37]. The minimum data criteria to be included in that study were that each dolphin would have at least 10 video observations and could be seen for the majority of those observations. This resulted in the 240-minute minimum criteria. The same criteria were used for the present study to remain consistent across manuscripts within the collection. Dolphins with less than 240 minutes of data recorded outside of formal training sessions (i.e., without human interaction) for both the 2018 and 2019 data collection periods were excluded from the analysis. For individuals with more than 240 minutes of data recorded in 2018 and 2019, the data from 2018 were used and the data from 2019 were excluded from the analysis. If only data from one period were more than 240 minutes, the period with more than 240 minutes were used in the analysis and data from the other period were excluded. Data were used from a single five-week data collection period because dolphins without qualifying data in both periods would have to be excluded entirely during the construction of the GEE models, further reducing the sample size. This maintained a large sample size and prioritized an investigation of variability across accredited facilities rather than exploring variation within individuals. A chi-square test of significance and an independent t-test were used to determine if the age and sex demographics of the dolphins in the final data set were statistically different than the original group of dolphins sampled.

Regression models were fitted using GEEs to allow for individual-level analyses and to account for facility ID. Facility ID was treated as a random effect with an independent correlation structure. Models were first built with univariate level predictors. Predictors with a sample size smaller than three were excluded from further analysis. Variables that correlated (*p* < 0.15) with the dependent variable were retained for building hierarchical models. The hierarchical model with the lowest quasi-likelihood under the independence model criterion (QIC) value and highest number of significant independent variables was selected as the final model. Analyses for the regression models were completed in SPSS 21. The final models that were considered with significant independent variables and the lowest QIC values are given in S2 File_ModelSelection_Lauderdale_HabitatUse.

## Results

Based on the minimum inclusion criteria, the final dataset included MTag deployments from 60 dolphins in 31 habitats. The sex (χ ^2^(1, N = 125) = 3.623, *p* = 0.057) and age (*t*(123) = 0.542, *p* = 0.589) distribution of the group of participants included in the analyses were not significantly different than the group composition prior to excluding dolphins that did not meet the inclusion criteria. Data were collected for 1053.35 hours (range: 255 to 2043 minutes per dolphin) during periods in which the dolphin was outside of formal training sessions. Of the participants, 57 were common bottlenose dolphins and three were Indo-Pacific bottlenose dolphins. Dolphins ranged from 3 to 44 years of age at the start of data collection (mean 16.48 ± 9.84 SD). The mean maximum depth was 5.63 m for the professionally managed ocean habitats and 8.78 m for professionally managed zoo/aquarium habitats. The mean time spent in the top third of their habitat was 80.01 ± 12.82% and the mean time spent in the bottom one third of the habitat was 4.03 ± 5.92%. The mean duration of a dive was 55.28 ± 15.42 sec.

Demographic and management factors associated with dive duration and vertical space usage (top/bottom third use) were evaluated. Univariate correlations where *p* < 0.15 were observed between dive duration and three enrichment variables (Table 2). Univariate correlations where *p* < 0.15 were observed between use of the top third of the habitat and one demographic variable, two enrichment variables, one training variables, and two habitat variables (Table 3). Univariate correlations *p* < 0.15 were observed between use of the bottom third of the habitat and two demographic variables, four enrichment variables, four training variables, and four habitat variables (Table 4). Descriptive statistics for independent variables considered for multivariate analysis are presented in Table 5.

**Table 2:** Univariate correlations between dive duration and independent variables.

Variables	Reference	n	Beta	*p*-value
***Demographic***				
Sex	Ref^a^ = Male	35	0.000	
	Female	25	-5.095	0.185
Age		60	-0.083	0.731
***Environmental Enrichment***				
Enrichment Diversity Index		60	-0.642	0.845
Enrichment Program Index		60	3.808	0.014*
Night Time Enrichment		60	0.365	0.642
Enrichment Schedule	Ref = Predictable	8	0.000	
	Semi-Random	44	-3.859	0.390
	Random	8	-11.863	0.099^
Frequency of New Enrichment	Ref = Year+	2	0.000	
	Twice a Year	16	-6.353	0.186
	Monthly/Weekly	42	-4.711	0.033*
***Training***				
Dolphin Presentations		60	-0.092	0.592
Interaction Programs		60	0.301	0.178
Training Duration		60	-0.047	0.849
Maximum Number Interaction Guests		60	0.007	0.974
Training Schedule	Ref = Predictable	29	0.000	
	Semi-Predictable	31	1.553	0.694
***Habitat Characteristics***				
Day Time Spatial Experience		60	-0.027	0.941
Night Time Spatial Experience		60	-0.103	0.785
24 Hour Spatial Experience		60	-0.083	0.824
Length		60	-0.010	0.902
Depth		60	0.248	0.648
Habitat Type	Ref = Zoo/Aquarium Habitat	35	0.000	
	Ocean Habitat	25	2.718	0.481
Number of Habitats		60	1.350	0.204
Social Management	Ref = Same Group	29	0.000	
	Split/Reunited at Night	20	6.104	0.528
	Rotated Subgroups	11	3.257	0.174
Neighboring Conspecifics	Ref = No Visual Access	35	0.000	
	Visual/Auditory Access	25	-4.687	0.246

^*p*-value < 0.15 used as threshold significant level for model building.

*p-value < 0.05.

a The reference value (Ref =) was the baseline value used when calculating univariate correlations with these binary variables.

**Table 3:** Univariate correlations between top third habitat use and independent variables.

Variables	Reference	n	Beta	*p*-value
***Demographic***				
Sex	Ref^a^ = Male	35	0.000	
	Female	25	-1.189	0.716
Age		60	-0.369	0.057^
***Environmental Enrichment***				
Enrichment Diversity Index		60	0.973	0.688
Enrichment Program Index		60	-2.456	0.117^
Night Time Enrichment		60	-0.270	0.647
Enrichment Schedule	Ref = Predictable	8	0.000	
	Semi-Random	44	4.650	0.284
	Random	8	7.519	0.216
Frequency of New Enrichment	Ref = Year+	2	0.000	
	Twice a Year	16	6.664	0.141^
	Monthly/Weekly	42	4.395	0.270
***Training***				
Dolphin Presentations		60	-0.026	0.870
Interaction Programs		60	-0.198	0.569
Training Duration		60	0.375	0.032*
Maximum Number Interaction Guests		60	0.161	0.338
Training Schedule	Ref = Predictable	29	0.000	
	Semi-Predictable	31	-3.010	0.358
***Habitat Characteristics***				
Day Time Spatial Experience		60	-0.160	0.560
Night Time Spatial Experience		60	0.008	0.978
24 Hour Spatial Experience		60	-0.088	0.751
Length		60	-0.026	0.640
Depth		60	0.241	0.486
Habitat Type	Ref = Zoo/Aquarium Habitat	35	0.000	
	Ocean Habitat	25	-6.810	0.025*
Number of Habitats		60	0.250	0.770
Social Management	Ref = Same Group	29	0.000	
	Split/Reunited at Night	20	-8.389	0.137^
	Rotated Subgroups	11	6.330	0.013*
Neighboring Conspecifics	Ref = No Visual Access	35	0.000	
	Visual/Auditory Access	25	4.047	0.214

^*p*-value < 0.15 used as threshold significant level for model building.

*p-value < 0.05.

a The reference value (ref =) was the baseline value used when calculating univariate correlations with these binary variables.

**Table 4:** Univariate correlations between bottom third habitat use and independent variables.

Variables	Reference	n	Beta	*p*-value
***Demographic***				
Sex	Ref ^a^ = Male	35	0.000	
	Female	25	3.144	0.052^
Age		60	0.169	0.082^
***Environmental Enrichment***				
Enrichment Diversity Index		60	0.859	0.484
Enrichment Program Index		60	2.349	0.013*
Night Time Enrichment		60	0.467	0.094^
Enrichment Schedule	Ref = Predicable	8	0.000	
	Semi-Random	44	-6.079	0.063^
	Random	8	-7.697	0.020*
Frequency of New Enrichment	Ref = Year+	2	0.000	
	Twice a Year	16	1.865	0.002*
	Monthly/Weekly	42	4.990	0.000*
***Training***				
Dolphin Presentations		60	-0.158	0.054^
Interaction Programs		60	0.438	0.034*
Training Duration		60	-0.205	0.030*
Maximum Number Interaction Guests		60	-0.153	0.041*
Training Schedule	Ref = Predictable	29	0.000	
	Semi-Predictable	31	-2.071	0.167
***Habitat Characteristics***				
Day Time Spatial Experience		60	-0.033	0.886
Night Time Spatial Experience		60	-0.074	0.765
24 Hour Spatial Experience		60	-0.052	0.827
Length		60	-0.046	0.032*
Depth		60	-0.135	0.231
Habitat Type	Ref = Zoo/Aquarium Habitat	35	0.000	
	Ocean Habitat	25	-3.013	0.024*
Number of Habitats		60	0.311	0.441
Social Management	Ref = Same Group	29	0.000	
	Split/Reunited at Night	20	3.036	0.103^
	Rotated Subgroups	11	-2.052	0.071^
Neighboring Conspecifics	Ref = No Visual Access	35	0.000	
	Visual/Auditory Access	25	-2.289	0.118^

^*p*-value < 0.15 used as threshold significant level for model building.

*p-value < 0.05.

a The reference value (Ref =) was the baseline value used when calculating univariate correlations with these binary variables.

**Table 5:** Descriptive statistics for the independent variables included in the multivariate modeling process.

Independent Variable	Reference	n	Mean	SD	Min	Max	Median
Age		60	16.48	9.84	3.00	44.00	14.00
Enrichment Program Index		60	0.02	1.01	-1.10	2.41	-0.17
Frequency of New Enrichment	Ref^a^ = Year+/Yearly	2	-	-	-	-	-
	Twice a Year	16	-	-	-	-	-
	Monthly/Weekly	42	-	-	-	-	-
Habitat Type	Ref = Zoo/Aquarium Habitat	35	-	-	-	-	-
	Ocean Habitat	25	-	-	-	-	-
Social Management	Ref = Same Group	29	-	-	-	-	-
	Split/Reunited at Night	20	-	-	-	-	-
	Rotated Subgroups	11	-	-	-	-	-

a The reference value (Ref =) was the baseline value used when calculating univariate correlations with these binary variables.

For dive duration, none of the multivariate models resulted in significance on all variables. Therefore, the final model only included the enrichment program index (Table 6) where longer dive durations were associated with higher enrichment program index values (β = 3.81, *p* = 0.01).

**Table 6:** Results for the final model examining dive duration.

Variable	Beta	Std error	Lower 95% CI	Upper 95% CI	*p*-value
(Intercept)	55.20	1.92	51.44	58.96	<0.01
Enrichment Program Index	3.81	1.55	0.76	6.85	0.01*

*p-value < 0.05.

The final multivariate model for top third habitat use included age, social management, and habitat type (Table 7). The model showed that top third habitat use decreased with age (β = -0.34, *p* = 0.02). Dolphins in professionally managed ocean habitats used the top third of the habitat less than those in professionally managed zoo/aquarium habitats (β = -8.32, *p* < 0.01). Dolphins that were managed in a group that was split into smaller subgroups during the day and were reunited into one group at night spent less time in the top third of the habitat than those who remained in a single group with consistent members at all times. (β = -9.51, *p* < 0.01).

**Table 7:** Results for the full multivariate model examining top third habitat use.

Variable	Beta	Std error	Lower 95% CI	Upper 95% CI	*p*-value
(Intercept)	91.50	3.20	85.23	97.78	<0.01
Age	-0.34	0.15	-0.64	-0.05	0.02*
Habitat Type: Zoo/Aquarium Habitat	-	-	-	-	-
Habitat Type: Ocean Habitat	-8.32	2.65	-13.52	-3.13	<0.01*
Social Management: Same Group	-	-	-	-	-
Social Management: Reunited	-9.51	3.07	-15.53	-3.48	<0.01*
Social Management: Rotated Subgroups	4.43	3.47	-2.37	11.24	0.20

*p-value < 0.05.

The final multivariate model for bottom third habitat use included interaction programs, new enrichment, social management, and enrichment program (Table 8). Dolphins who participated in a larger mean number of interaction programs used the bottom third of the habitat more often (β = 0.32, *p* = 0.04). Dolphins receiving new enrichment on a monthly/weekly schedule also used the bottom third of the habitat more often than those receiving new enrichment on a yearly/year+ schedule (β = 4.97, *p* < 0.01). Dolphins that were managed as subgroups with rotating members but were never united as one group spent less time in the bottom third of the habitat than those who remained in a single group with consistent members at all times (β = -2.34, *p* = 0.05). Dolphins provided with enrichment programs that had higher index values used the bottom third of the habitat more often (β = 2.22, *p* < 0.01).

**Table 8:** Results for the full multivariate model examining bottom third habitat use.

Variable	Beta	Std error	Lower 95% CI	Upper 95% CI	*p*-value
(Intercept)	1.81	1.06	-0.27	3.89	0.09
Interaction Programs	0.32	0.15	0.02	0.62	0.04*
Frequency of New Enrichment: Yearly/Year+	-	-	-	-	-
Frequency of New Enrichment: Twice per year	1.23	1.10	-0.93	3.39	0.27
Frequency of New Enrichment: Monthly/Weekly	4.97	1.20	2.62	7.32	<0.01*
Social Management: Same Group	-	-	-	-	-
Social Management: Reunited	1.78	1.44	-1.05	4.61	0.22
Social Management: Rotated Subgroups	-2.34	1.22	-4.74	0.06	0.05*
Enrichment Program Index	2.22	0.78	0.70	3.74	<0.01*

*p-value < 0.05.

## Discussion

This research represents the first time that bio-logging devices have been used to compare the habitat use and long-scale diving behavior of bottlenose dolphins under professional care at a large number of accredited facilities. The findings are strengthened by the considerable number of individuals and habitats represented in the data. These efforts are complementary in the continual research and understanding of normal dolphin activity and physiology to better apply the comparison of the biological components (i.e., drag) of dolphin movement within a habitat. The results suggested that demographic, environmental enrichment, training, and habitat characteristics impacted dive durations and habitat use in various ways.

The mean dive duration (55.28 sec) was comparable or longer than the dives of wild dolphins. Mean dive durations for wild coastal bottlenose dolphin populations range from 25 to 55 sec [38–40] and 22 sec in a previous report for dolphins under professional care in a single habitat [41]. Dive duration was not related to habitat characteristics (i.e., volume, depth, or length) in the final model. The only statistically significant variable in the final model for dive duration was the enrichment program index (a synthesized score created based on frequency with which facilities engaged in several evaluative aspects of their enrichment programs). This finding suggested that features of environmental enrichment program (e.g., setting goals and evaluating enrichment) were not only related to activity levels [42], but also were related to longer diving bouts. This may be due, in part, to the effort by facilities to develop environmental enrichment that sinks to the bottom of the habitat and are designed to stimulate use of the full depth of the environment [43,44].

Consistent with previous investigations of habitat use of dolphins under professional care [45], the results in the present study suggested that dolphins prefer to inhabit the upper portions of the water column. It is important to note that the habitat use described in the present study was referring to the top and bottom portion of the habitat and not absolute depth. Rather than comparing absolute depth, the goal was to understand how dolphins used the habitats available to them. In a previous study, when given the option to select their environment from several areas with varying depths, bottlenose dolphins spent more time in areas with shallow and moderate depths [27]. Similarly, coastal bottlenose dolphins were most often found at comparable depths [21,22]. Previous findings indicated that when inhabiting deeper water (> 150 m), wild dolphins continued to primarily swim within the top 5 m of habitat [23]. Dolphins in the present study spent the majority of their time in the upper portions of the water column and had a mean dive duration longer than reports of wild dolphins’ dives [38–40]. The lack of time spent in deeper areas documented in previous studies and the underutilization of the bottom portions of the habitats in the present study suggests that current depths of many habitats for bottlenose dolphins may be sufficient in meeting their behavior and welfare needs for movement. However, the present study included both *Tursiops truncatus* and *Tursiops aduncus* subspecies. Future research should further investigate if the two subspecies exhibit differences in habitat use as a result of size or behavior under professional care. In addition, the length of the individual, interactions with trainers, high energy behaviors, and interactive cohort behavior may be additional considerations when determining appropriate habitat depths for specific facilities or groups of dolphins.

Age, social management, and habitat type were variables included in our final model for top third habitat use. As dolphins aged, they used the top third of the habitat less often. One explanation may be that older animals learned to reduce their activity levels by diving to deeper depths where drag is reduced [44]. Similarly, previous reports found that dolphins heavily favored swimming in the bottom two thirds of the habitat when engaging in slow, resting swimming patterns [46]. Other results from the Cetacean Welfare Study suggested that overall dynamic body acceleration (ODBA; a proxy for energy expenditure) also reduced with age [37,42]. Future analysis that compares swimming gait and the energetic costs associated with swimming at different depths could provide further insight into these results.

Social management was also related to habitat use. Dolphins managed as a split/reunited group utilized the top third of the habitat less often than dolphins managed as a single group. Dolphins in the split/reunited category were managed in a group that was split into smaller subgroups during the day and were reunited into one group at night. The reasoning behind this finding is unclear and would be a favorable topic for future research. Dolphins in professionally managed ocean habitats swam in the top third of the habitat less often than dolphins in professionally managed zoo/aquarium habitats. This may have been due to animals having access to natural flora and fauna in the ocean environment. For example, some professionally managed ocean habitats may have included additional underwater opportunities for interaction with natural substrates and grasses as well as species of fish that may have entered and exited the habitat. It is also possible that this may have been attributed to the mean maximum depth of professionally managed zoo/aquarium habitats in this study being 3.15 m deeper than professionally managed ocean habitats and, as a result, each third of the habitat was a smaller relative distance. That is, the dolphins may have a favorable absolute depth to swim at, but because the definitions for top and bottom thirds were in a relative scale at each facility, the preferred absolute depth could be categorized within different thirds depending on the facility. For example, a dolphin swimming at three meters deep may have been considered in the middle third of a professionally managed ocean habitat but in the top third of a professionally managed zoo/aquarium habitat. In addition, use of the top third of the habitat may have been related to the amount of floating and sinking enrichment provided to the dolphins and that dolphins received food from animal care staff at the surface. Use of the top third of the habitat also may have been related to the proximity of animal care staff and/or guests throughout the day. It is possible that dolphins spent time observing animal care staff and/or guests which may have impacted their behavior resulting in more time spent in the top third of the habitat. Future research should investigate how floating and sinking enrichment as well as the proximity of animal care staff and/or guest are related to water column use.

Bottom third habitat use was related to the mean number of interaction programs each week, frequency of new enrichment, social management, and the enrichment program. Dolphins who participated in a larger mean number of interaction programs used the bottom third of the habitat more often, but the magnitude of change in bottom third habitat use per interaction program was small. As most facilities with professionally managed ocean habitats offered interaction programs rather than presentations, it is possible that this was an artifact of professionally managed ocean habitats being an average of 3.15 m shallower than professionally managed zoo/aquarium habitats in this study. Again, if habitats tended to be shallower, they may have been more likely to use the middle or bottom third of the habitat based on a preferred absolute depth. Dolphins receiving new enrichment on a monthly/weekly schedule (compared to a yearly/year+ schedule) and enrichment programs with higher index values used the bottom third of the habitat more often. One possible explanation is that more complex and continually updated enrichment programs may have incorporated more sinking or underwater enrichment that promoted use of the bottom third of the habitat. Dolphins receiving new enrichment on a monthly/weekly schedule also had higher ODBA values, rates of group active behaviors, and rates of interactions with conspecifics when compared to dolphins receiving enrichment on a yearly/year+ schedule [37,42], suggesting that these variables may have been associated. Dolphins managed in rotating subgroups used the bottom third of the habitat less often than those managed in a consistent group. A possible explanation for this relationship is unknown and future research should work to further understand this finding.

The variety of factors that were related to dive duration and habitat use highlight the complexity of the topic. The present study is unique in both its use of bio-loggers to quantify behavior and in terms of the scale and breath of the participating dolphin and facilities sample sizes. Demographic, environmental enrichment, training, and habitat characteristics were all influential in how bottlenose dolphins used their habitats. The results suggested that management practices had a predominate role in how dolphins interacted with their environment when compared to the physical characteristics of the habitat. Future research should focus on understanding the impacts of social management and further assessing the effects of habitat dimensions on habitat use. In addition, research should investigate optimal habitat size while considering group size, high energy behaviors, cohort interactions, and the type and location of trainer interactions. In terms of management, future resources should be directed towards continuing to develop comprehensive enrichment programs.

## Supporting information

S1 File Appendix1 Lauderdale habitat use. (XLSX)

S2 File Model selection Lauderdale habitat use. (XLSX)

S3 File Descriptive statistics Lauderdale habitat use. (XLSX)

S4 File Striking image Lauderdale habitat use. (TIFF)
