# Supplementary figures and images for: Habitat characteristics and animal management factors associated with habitat use by bottlenose dolphins in zoological environments
Source: PLoS One. 2021 Aug 30;16(8):e0252010. doi: 10.1371/journal.pone.0252010 (PMC8404980; doi:10.1371/journal.pone.0252010)

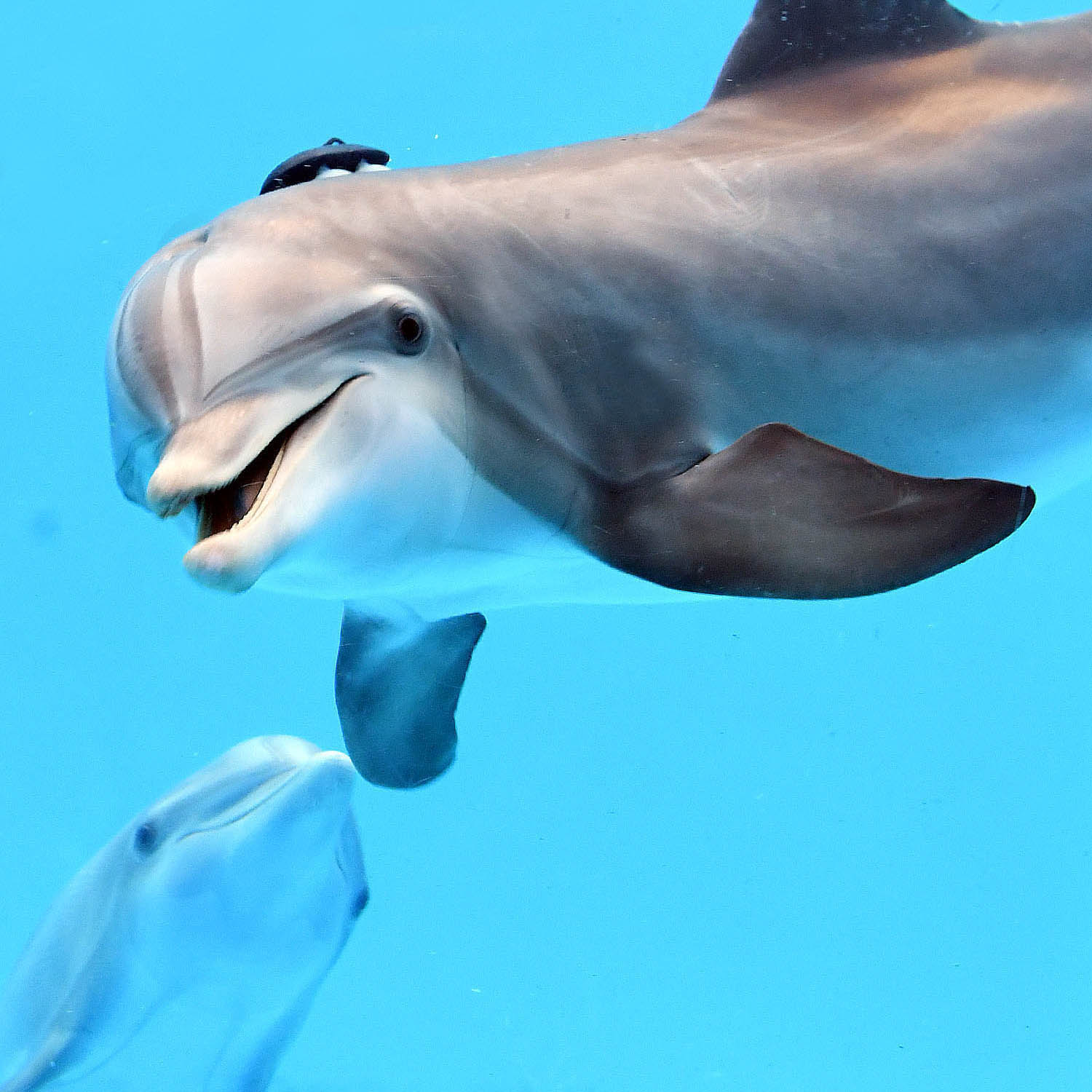

Supplement: S4 File — (TIFF) [file pone.0252010.s004.tiff]
